# The association between allergy and sinusitis: a cross-sectional study based on NHANES 2005–2006

**DOI:** 10.1186/s13223-021-00642-2

**Published:** 2021-12-25

**Authors:** Song Li, Chu-Jin Zhao, Hong-Li Hua, Yu-Qin Deng, Ze-Zhang Tao

**Affiliations:** 1 Department of of Otolaryngology-Head and Neck Surgery, Renmin Hospital of Wuhan University, 238 Jie-Fang Road, Wuhan, 430060 Hubei People’s Republic of China; 2 Department of of Otolaryngology-Head and Neck Surgery, Xianning Central Hospital of Hubei Province, 228 Jingui Road, Xian’an, Xianning, 437000 Hubei People’s Republic of China

**Keywords:** Allergens, Sinusitis, Allergic rhinitis, Serum total IgE

## Abstract

**Background:**

The relationship between allergies and sinusitis, though extensively studied, remains poorly defined. While several studies proposed a cause-and-effect relationship between allergy and chronic sinusitis, several others reported the lack of any existing association. This study aimed to investigate the relationship between allergy and sinusitis.

**Methods:**

We conducted a cross-sectional study using a representative sample of the US population from the National Health and Nutrition Examination Survey 2005‒2006 (n  = 7244). A self-reported allergy questionnaire and total and allergen-specific IgE levels were used for analysis. Participants were divided into positive and negative allergy symptoms groups (PAS, NAS, respectively) to eliminate the influence of allergy symptoms on the apparent incidence of sinusitis. Pearson’s chi-square test and the linear regression analysis using Durbin Watson test were used for statistical analysis.

**Results:**

Sinusitis incidence in the PAS group (22.4%; 521/2327) was significantly higher than that in the NAS group (7.1%; 348/4917) [odds ratios (OR)  = 3.788, 95% confidence interval (CI) 3.272‒4.384, *P*  < 0.001]. sinusitis incidence in non-sensitized and sensitized groups was not statistically different. After controlling for allergy symptoms, there was a negative correlation between sensitization status and the occurrence of sinusitis in the PAS group (OR  = 1.407, 95% CI 1.156‒1.711, *P*  < 0.01). Increase in serum total IgE levels correlated with decrease in incidence of sinusitis in both PAS and NAS groups. sinusitis incidence was significantly reduced in the PAS group in participants sensitized to allergens such as cockroaches, ragweed, ryegrass, Bermuda grass, oak, birch, and thistle.

**Conclusion:**

Allergy is related to sinusitis incidence. It is likely that sensitization status could reduce the incidence of sinusitis, albeit in an antigen-specific manner.

## Introduction

The pathogenesis of chronic sinusitis is complex and multifactorial. In the past, chronic sinusitis was characterized as a disease caused by sinus ostia obstruction. However, our understanding of this disease process has evolved considerably, and it is widely accepted that other chronic inflammatory processes play an important role in the occurrence and development of chronic sinusitis [1]. The relationship between allergies and chronic sinusitis is a topic that has been extensively studied, but the association between these conditions remains poorly defined. Some studies have proposed a cause-and-effect relationship between allergy and chronic sinusitis [2–4], while other studies did not find any such association [5–7]. Wilson’s review of this topic, published in 2014, was a representative summary. The study reviewed 24 studies, of which 18 explored the relationship between allergy and chronic rhinosinusitis with nasal polyps. Eleven of these showed a correlation, while seven showed no correlation between these conditions. In addition, nine studies investigated the relationship between allergies and chronic rhinosinusitis without nasal polyps, of which four showed a correlation and five indicated no correlation [8]. Tantilipikorn [9] provided a recent update on this topic in 2020, in which 11 studies were included in a qualitative analysis of the association between systemic allergy and chronic rhinosinusitis. Of the 11 studies, four showed an association, three were inconclusive, and four did not show any association. Therefore, more studies on this topic are urgently needed to clarify the relationship between allergic rhinitis and sinusitis.

The National Health and Nutrition Examination Survey (NHANES) is a set of studies that evaluated the health and nutritional status of adults and children in the United States [10]. In the NHANES 2005‒2006 survey, information on serum total IgE and 19 allergen-specific IgEs, as well as investigations of allergy symptoms and sinus infection, were included. The NHANES adopted a stratified multistage sampling design to obtain a representative sample of USA residents, which provides a reliable database for investigating the relationship between allergy and sinusitis. In this study, we evaluated the relationship between allergic rhinitis and sinusitis by studying the relationship between sensitization status, allergy symptoms, and sinusitis; the relationship between total serum IgE and sinusitis, and the relationship between 19 allergen-specific IgEs and sinusitis, using this population-based dataset.

## Materials and methods

### Data sources

The data used in this study were obtained from the National Center for Health Statistics (NCHS) for NHANES 2005‒2006, which was the only year in which data on allergy and sinus infection were collected. Since all NHANES data were de-identified, the study did not require the approval of the institutional board review committee or the informed consent of participants.

A total of 10,348 participants were included in the NHANES 2005‒2006. The serum total IgE, antigen-specific IgE, allergy symptom questionnaire, and demographic information data were downloaded from the database for subsequent analysis. Participants with missing data were excluded from the study.

### Definition of sensitization

Serum samples were analyzed for total and allergen-specific IgE using the Pharmacia Diagnostics ImmunoCAP 1000 System (Kalamazoo, MI). A detailed description of the laboratory methods used can be found on the NHANES 2005‒2006 web page [11]. Nineteen allergen-specific IgEs were assessed. According to the American Clinical and Laboratory Standards Institute guidelines, we defined sensitization as when the level of at least one of the 19 allergens was not less than 0.35 kU/L [12].

### Definition of allergy symptoms and sinusitis

Information on allergy symptoms and sinusitis was obtained from the 2005‒2006 allergy questionnaire. A detailed description of the questionnaire instruments can be found on the NHANES 2005‒2006 web page [13]. Participants who answered yes to “During the past 12 months, (have you/has SP) had a problem with sneezing, or a runny, or blocked nose when (you/s/he) did not have a cold or the flu?” “Has a doctor or other health professional ever told (you/SP) that (you have/SP s/he has) hay fever?” were defined as having allergy symptoms. Participants who answered yes to “During the past 12 months, did a doctor or other health professional tell (you/SP) that (you have/SP s/he has) a sinus infection?” were defined as those with sinusitis.

### Study design

Our research included three aspects: the effect of sensitization on sinusitis, the effect of serum total IgE on sinusitis, and the effect of allergen-specific IgEs on sinusitis. When nasal hypersensitivity symptoms appear, the mucosa of the nasal cavity is inflamed and edematous, which leads to poor drainage of the sinus ostia and increases the risk of sinusitis. Therefore, when we analyzed these three issues, we divided participants into two cohorts according to whether they had allergy symptoms (a positive cohort with allergy symptoms and a negative cohort without allergy symptoms) for independent analysis. When analyzing the effect of serum total IgE on sinusitis, it was necessary to group participants according to the level of total serum IgE. With consideration of balancing the sample size in each group, we divided the participants into seven groups defined by total serum IgE:  < 10 kU/L, 10.01‒20 kU/L, 20.01‒40 kU/L, 40.01‒80 kU/L, 80.01‒160 kU/L, 160.01‒320 kU/L, and  > 320 kU/L. The sinusitis rates in these seven groups of participants were compared.

### Statistical analysis

Pearson’s chi-square test was used to analyze the impact of sensitization status and of 19 antigen-specific IgEs on sinusitis. When the theoretical frequency was less than 5 or the sample size was less than 40, the chi-square test with continuous correction was used. Participants were divided into seven groups in the study of the effect of serum total IgE on sinusitis, as defined above, and the linear regression analysis using Durbin Watson test was used to conduct trend analysis. All statistical assessments were two-sided, with a significance level of *P*  < 0.05. Analyses were performed using IBM SPSS Statistics 24 (IBM, Armonk, NY) and Python version 3.6.

## Results

A total of 7244 participants, including 3519 males (6–85 years, 35.2 ± 23.1 years) and 3725 females (6–85 years, 34.1 ± 22.2 years) were included after excluding participants with missing questionnaire data and serum test data.

**Fig. 1:**
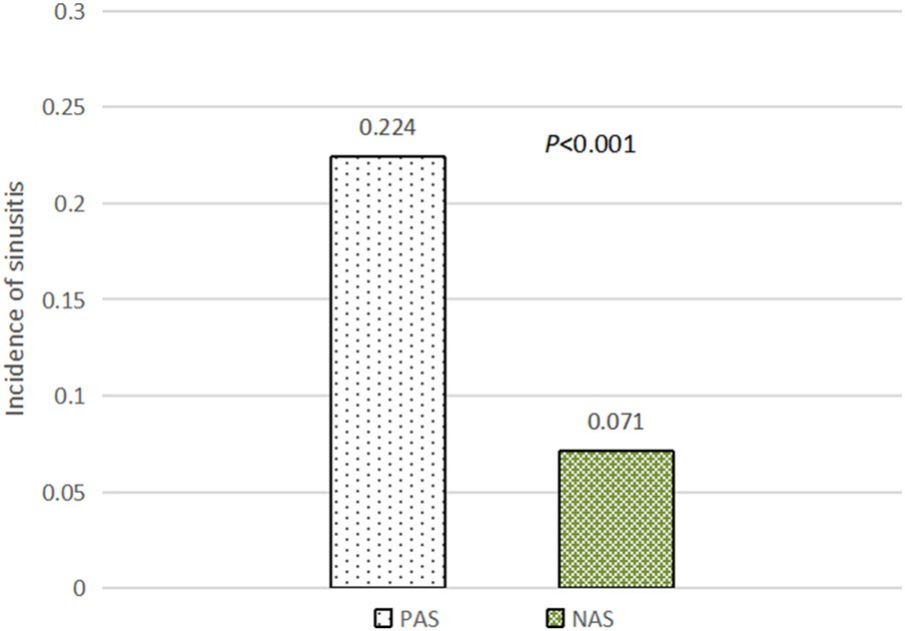
The incidence of sinusitis in the PAS and NAS groups. *PAS* positive allergy symptoms group; *NAS* negative allergy symptoms group

**Fig. 2:**
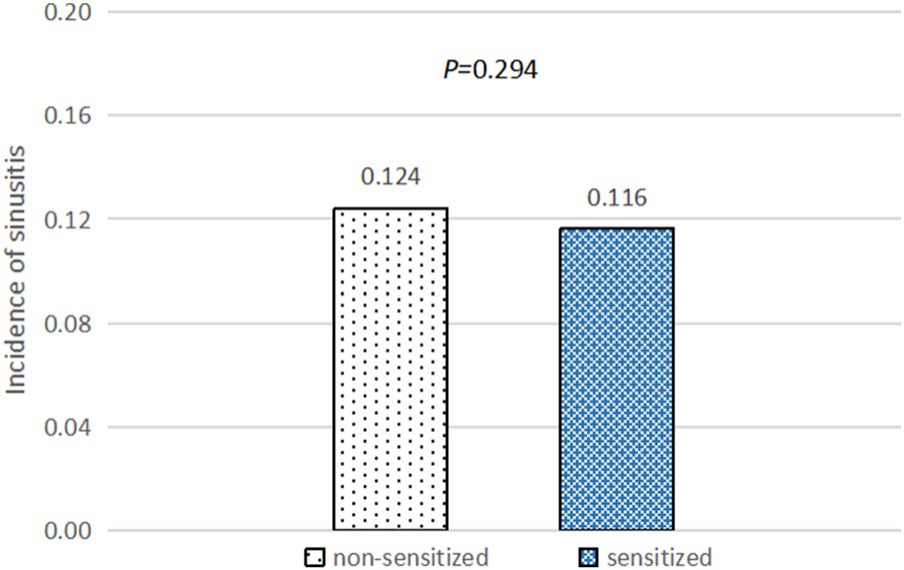
The incidence of sinusitis in the non-sensitized and sensitized groups

Participants were divided into a positive allergy symptoms group (PAS group) and a negative allergy symptoms group (NAS group) based on allergy symptoms. The incidence of sinusitis in the PAS group (22.4%, 521/2327) was significantly higher than that in the NAS group (7.1%, 348/4917) [odds ratio (OR)  =  3.788, 95% confidence interval (CI) 3.272‒4.384, *P * < 0.001] (Fig. 1), which suggests that allergy symptoms could significantly increase the risk of sinusitis. Participants were then divided into sensitized and non-sensitized groups according to whether they showed sensitization when allergy symptoms were not controlled. The incidence of sinusitis in the sensitized and non-sensitized groups was 11.6% (405/3497) and 12.4% (464/3747), respectively, suggesting that there was no significant difference according to sensitization (*P*  = 0.294) (Fig. 2).

**Fig. 3:**
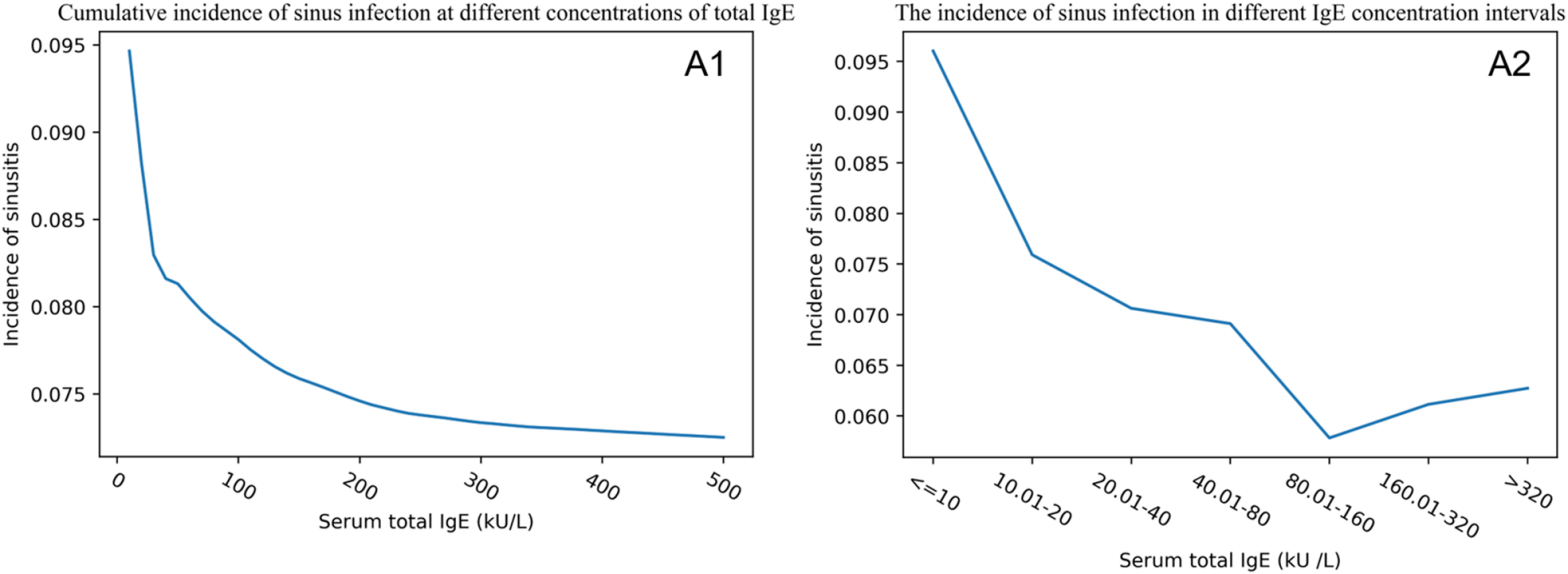
The relationship between serum total IgE and sinusitis in the NAS group. *NAS* negative allergy symptoms group. **A1** The cumulative incidence of sinusitis when IgE is less than 500 kU/L. For example, the graph depicts that the cumulative incidence of sinusitis for all participants with total IgE less than 200 kU/L is approximately 7.5%. **A2** The incidence of sinusitis in the corresponding concentration intervals of IgE

**Fig. 4:**
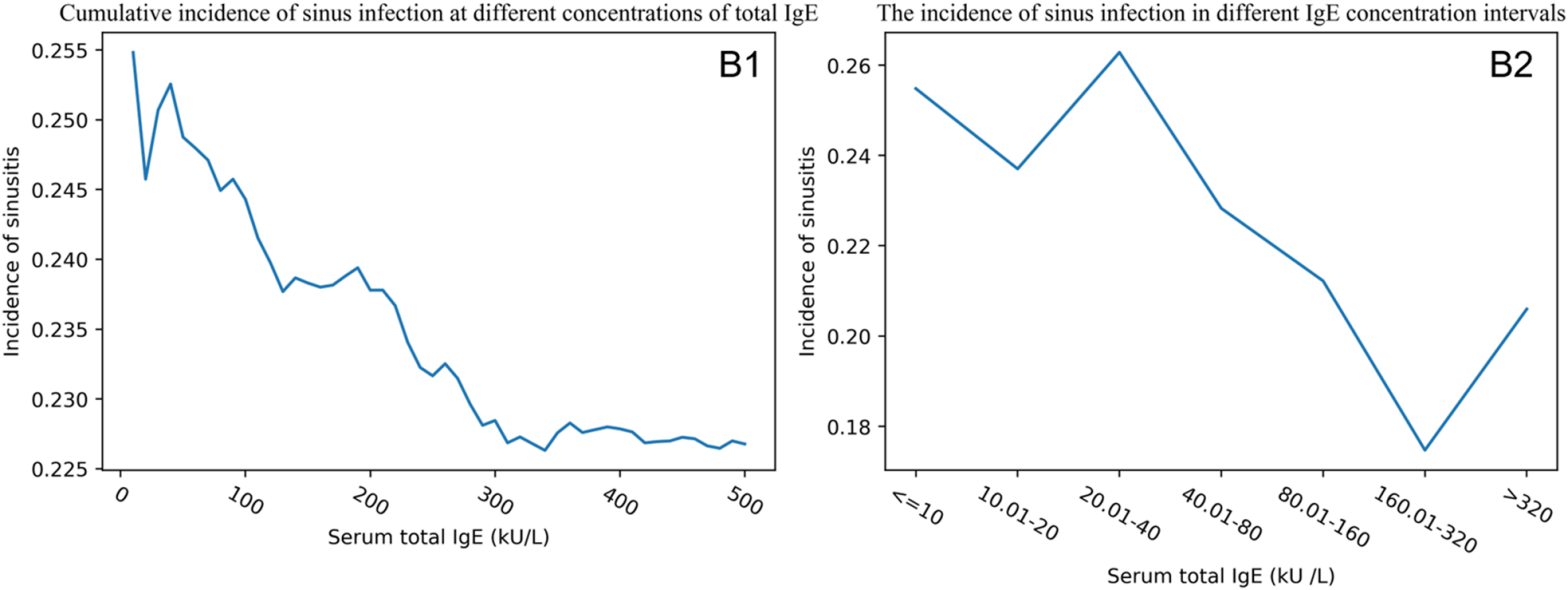
The relationship between serum total IgE and sinusitis in the PAS group. *PAS* positive allergy symptoms group

The cumulative incidence of sinusitis with serum total IgE within 500 kU/L was evaluated as an increased unit for every 10 kU/L. With the increase in serum total IgE concentration, the cumulative incidence of sinusitis in the NAS group gradually decreased from 9.5 to 7.2%, and the cumulative incidence of sinusitis in the PAS group gradually decreased from 25.5 to 22.7%. To explore the specific incidence of sinusitis with different serum total IgE levels, the participants were divided into seven subgroups. The incidence of sinusitis for the seven subgroups,  ≤ 10 kU/L, 10.01–20 kU/L, 20.01–40 kU/L, 40.01–80 kU/L, 80.01–160 kU/L, 160.01–320 kU/L,  > 320 kU/L), in the NAS group (R^2^  = 0.711, *P*  = 0.017) was 9.6% (63/656), 7.6% (52/685), 7.1% (63/892), 6.9% (64/926), 5.8% (41/709), 6.1% (31/507), and 6.3% (34/542), respectively, while in the PAS group (R^2^  = 0.803, *P*  = 0.006) was 25.5% (66/259), 23.7% (64/270), 26.3% (92/350), 22.8% (92/403), 21.2% (73/344), 17.5% (58/332), and 20.6% (76/369), respectively (Figs. 3,  4). The results indicated that the incidence of sinusitis decreased with the increase in serum total IgE level in both the NAS group and PAS group.

**Fig. 5:**
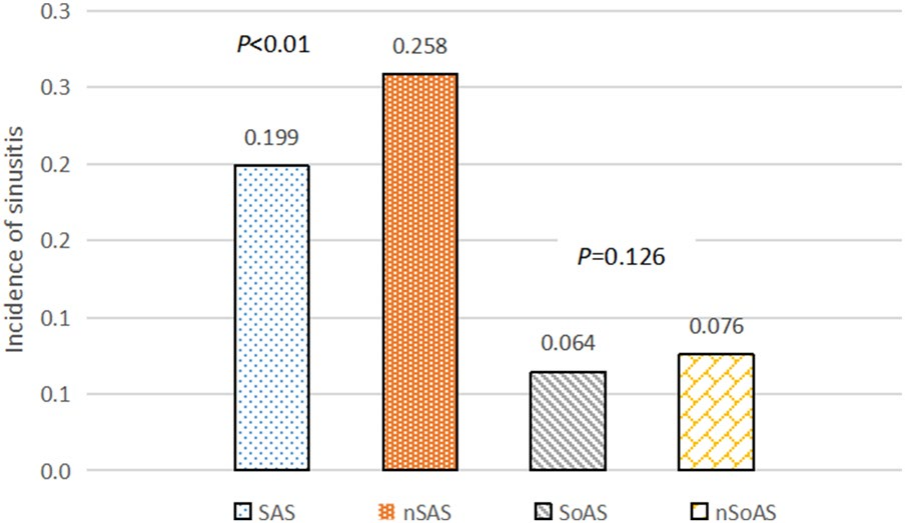
The incidence of sinusitis in the SAS, nSAS, SoAS, and nSoAS groups. *SAS* sensitization group with allergy symptoms; *nSAS* non-sensitized group with allergy symptoms; *SoAS* sensitization group without allergy symptoms; *nSoAS* non-sensitized group without allergy symptoms

The allergy symptoms were controlled, and the PAS and NAS groups were separately divided into two subgroups: the sensitized group and the non-sensitized group, namely the sensitization group with allergy symptoms (SAS), the non-sensitized group with allergy symptoms (nSAS), the sensitization group without allergy symptoms (SoAS), and the non-sensitized group without allergy symptoms (nSoAS). The incidence of sinusitis was 19.9% (266/1340), 25.8% (255/732), 6.4% (139/2157), and 7.6% (209/2760) in the SAS, nSAS, SoAS, and nSoAS groups, respectively. There was a significant difference in the incidence of sinusitis between the SAS and nSAS groups (OR = 1.407, 95% CI 1.156‒1.711, *P* < 0.01). However, there was no significant difference in the incidence of sinusitis between the SoAS and nSoAS groups (*P* = 0.126) (Fig. 5).

**Fig. 6:**
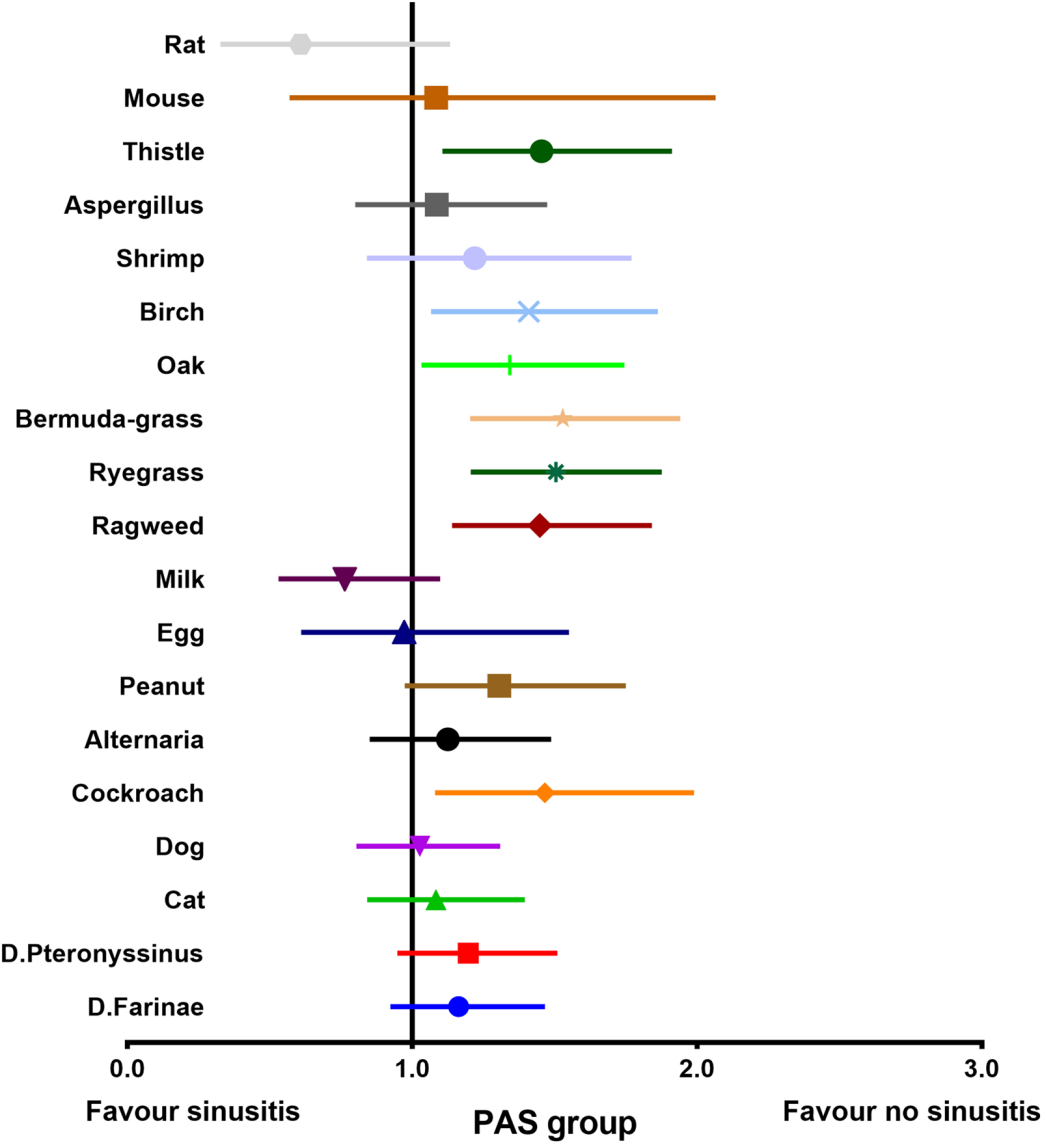
The relationship between 19 specific antigens sensitization and sinusitis in PAS group. *PAS* positive allergy symptoms group

**Fig. 7:**
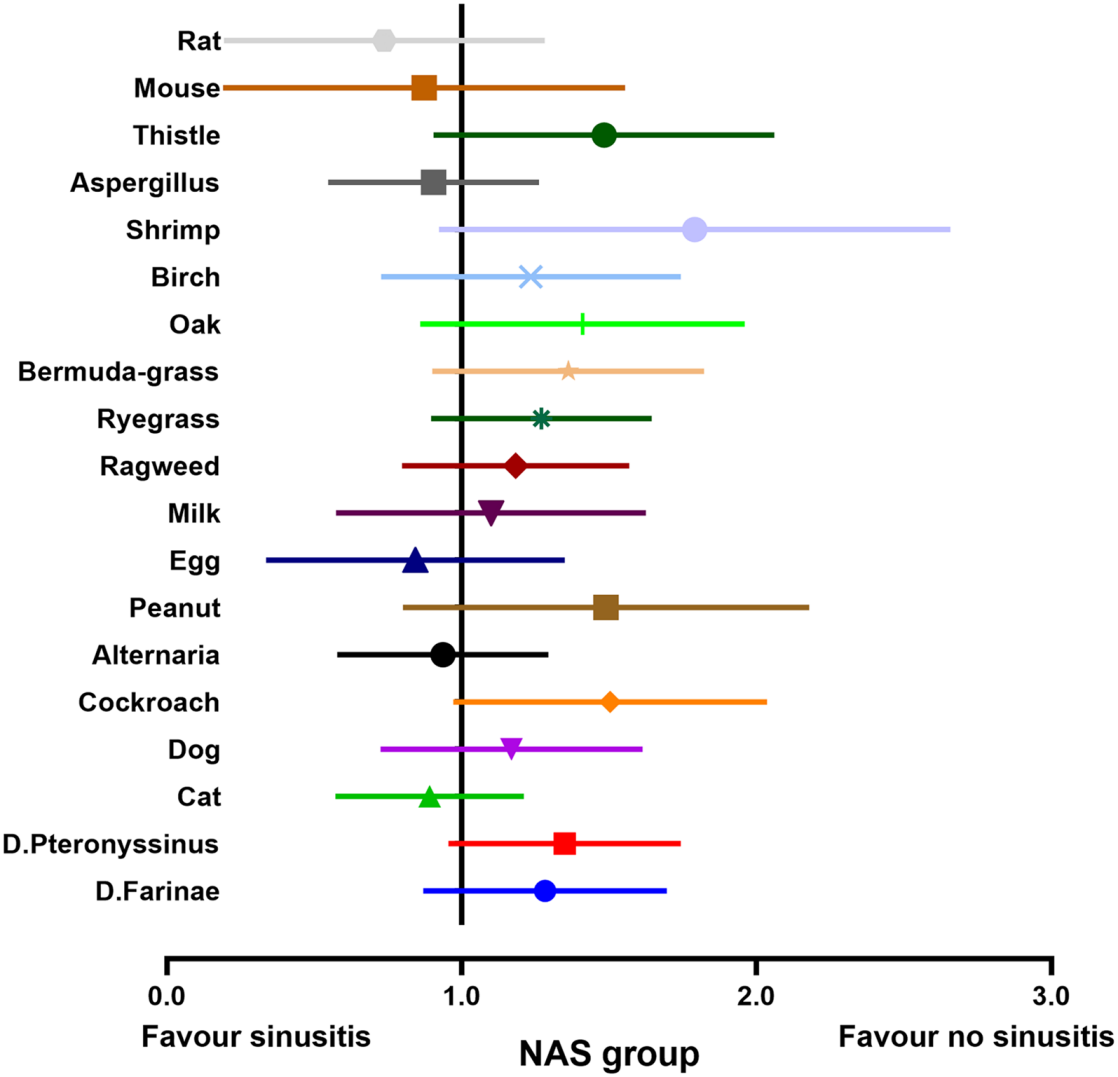
The relationship between 19 specific antigens sensitization and sinusitis in NAS group. *NAS* negative allergy symptoms group

These results indicate a negative correlation between the sensitization status and the occurrence of sinusitis. In order to clarify whether all 19 types of antigen-specific IgE in serum had a negative correlation with sinusitis, we analyzed each allergen independently in the PAS and NAS groups. In the PAS group, the incidence of sinusitis was significantly lower when the participants were sensitized to cockroach (OR  =  1.466, 95% CI 1.080‒1.990, *P*  = 0.0168), ragweed (OR  = 1.448, 95% CI 1.140‒1.841, *P*  = 0.0029), ryegrass (OR  = 1.504, 95% CI 1.205‒1.876, *P*  = 0.0003), Bermuda grass (OR  = 1.528, 95% CI 1.203‒1.941, *P*  = 0.0006), oak (OR  = 1.342, 95% CI 1.032‒1.745, *P*  = 0.0026), birch (OR  = 1.409, 95% CI 1.066‒1.862, *P * = 0.0188), and thistle (OR  = 1.454, 95% CI 1.106‒1.912, *P*  = 0.0086) (Fig. 6). However, none of the allergens showed a significant effect on the incidence of sinusitis in the NAS group (Fig. 7).

## Discussion

The mechanism underlying chronic rhinosinusitis is multifactorial. Generally speaking, local tissue aberrations, such as epithelial tissue hypersensitivity, disruptions of innate immunity, the presence of bacterial colonization and biofilm, and genetic and environmental factors may all play a role in disease pathogenesis [14]. Although the relationship between allergy and sinusitis remains controversial, there should be an implicit internal relationship between them considering that both conditions have a pathological basis related to type 2 inflammation [15]. Patients with allergy symptoms have nasal mucosal edema, increased secretions, and impaired mucociliary function, which may lead to sinus obstruction. Blockade of sinuses with poor drainage can cause bacterial infection, which eventually leads to sinusitis. However, the relationship between allergy symptoms and sinusitis and allergies is an indirect relationship. In fact, whether there is a direct connection between them, such as at the level of cytokines, is of greater interest. Because nasal allergy symptoms have a significant impact on the incidence of sinusitis, we divided the participants into PAS and NAS groups when we further explored the relationship between allergy and sinusitis. Since NHANES 2005‒2006 only had limited allergy-related data and it was infeasible to explore cytokine levels, we focused on the impact of total IgE and antigen-specific IgE on sinusitis, for which the database information was adequate. In this study, we found that individuals with allergy symptoms had a sinusitis rate 3.1 times higher than those without allergy symptoms (22.4 vs. 7.1%). However, the incidence of sinusitis in the non-sensitized and sensitized groups was not statistically significantly different. After controlling for allergy symptoms, we found a significant negative correlation between sensitization status and the occurrence of sinusitis in the PAS group (OR  = 1.407, 95% CI 1.156‒1.711, *P*  < 0.01). The incidence of sinusitis in the PAS group was significantly reduced in participants sensitized to cockroaches, ragweed, ryegrass, Bermuda grass, oak, birch, and thistle. Moreover, with the increase in serum total IgE level, the incidence of sinusitis decreased in both the PAS (*P*  = 0.017) and NAS groups (*P * = 0.006).

The role of serum total IgE in the evaluation and diagnosis of allergic diseases has been controversial. Several studies have demonstrated that serum total IgE plays a relevant role in the evaluation and diagnosis of allergic rhinitis. Jacobs et al. [16] reported that measuring total IgE was useful in diagnosing allergic rhinitis, particularly at levels higher than 100 IU/mL. Li et al. [17] observed that total IgE levels were higher in individuals with allergic rhinitis than in those with non-allergic rhinitis, in a retrospective study. In contrast, some studies have reported negative results when evaluating the association between serum total IgE and AR. Tu et al. [18] demonstrated total IgE levels had insufficient diagnostic accuracy to detect allergic diseases, regardless of the cutoff value used. Tay et al. [19] conducted a retrospective analysis in patients with high total IgE levels (> 1000 IU/mL) and concluded that elevated IgE levels are of limited clinical value in allergic rhinitis. In addition to allergic diseases, elevated serum total IgE levels can also be seen in many other diseases, including parasitic and viral infections, primary immunodeficiency (such as high IgE syndrome), and malignant tumors (such as Hodgkin’s lymphoma). Therefore, elevated serum total IgE levels are not specific to allergic conditions.

The relationship between serum total IgE levels and sinusitis is also controversial. Some studies have reported that high serum total IgE level is a risk factor for severe chronic sinusitis [20, 21]. Particularly the staphylococcal superantigens serum total IgE. Van et al. suggested a strong proinflammatory and IgE-inducing effect of Staphylococcus aureus enterotoxins in nasal tissue [22]. However, other studies concluded that the association between systemic atopic status and chronic sinusitis severity is weak, and that chronic sinusitis is an inflammatory disease that occurs independently of systemic IgE-mediated pathways [23]. According to Zhang et al. in patients with chronic rhinosinusitis with nasal polyps, the reactivity of tissue mast cells to allergen exposure and the presence of specific IgE to inhalant allergens corresponded in tissues, but not in sera, which was not the case in patients with allergic rhinitis [24]. In our study, we found that serum total IgE levels were negatively correlated with the incidence of sinusitis. Further studies with larger samples are needed to elucidate this relationship.

With regard to the relationship between allergen sensitization and sinusitis, we obtained interesting results after controlling for allergy symptoms. There was a negative correlation between sensitization status and the incidence of sinusitis in the PAS group, while there was no such association in the NAS group. We then investigated whether this negative correlation was antigen-specific. Analysis of 19 types of allergens revealed that the negative-correlated-antigens seemed to be species aggregated. We propose two hypotheses to explain the negative relationship in the PAS group but its absence in the NAS group. The first hypothesis is based on the lack of subdivision of allergy symptoms of participants into perennial and seasonal allergies. As perennial allergic rhinitis involves a longer period of sinus ostia blockage than seasonal allergic rhinitis, it is more likely to become a high-risk factor for sinusitis. Many studies have suggested that perennial allergens play a more significant role in the etiology of sinusitis than seasonal allergens [14, 25, 26]. Considering that most of these negative-correlated antigens are plant antigens, which are mainly spread by pollen and primarily cause seasonal allergies, this hypothesis seems to be reasonable. However, this hypothesis cannot explain the effect of sensitization to cockroaches, which is a typical perennial allergy. Considering that the *P* value of the relationship between sensitization to cockroaches and sinusitis in the NAS group was 0.0546, which approached statistical significance, this negative correlation result cannot be explained by the differences related to perennial and seasonal allergies. Another hypothesis is that sensitization to different allergens has different effects on the process of epithelial remodeling. The epithelial inflammatory response to allergens is a key feature of allergic rhinitis. Upon allergen exposure, there is significantly higher infiltration of inflammatory cells and increased levels of cytokines (such as IL-4, IL-5, and IL-13) in the nasal epithelium of allergic patients than in non-allergic patients. This inflammatory response translates into mucosal edema, autonomic neural stimulation, and increased mucosal secretions, which manifest as the hallmark symptoms of nasal obstruction, pruritus, sneezing, rhinorrhea, and smell loss in severe cases. In contrast to the epithelial inflammatory response, epithelial remodeling is a key feature of chronic rhinosinusitis (epithelial hyperplasia, goblet cell hyperplasia, and squamous metaplasia) and asthma (epithelial desquamation, subepithelial fibrosis, and smooth muscle hypertrophy) [27–29]. Remodeling in allergic rhinitis is less marked. We considered whether it could be possible that, after being sensitized to negative-correlated-antigens, the nasal epithelium would be inhibited from remodeling, while not being sensitized to a “conventional” allergen. There may be different pathways of epithelial inflammation in the human body after sensitization by different allergens, which could lead to different outcomes in epithelial cells. Birch pollen has been found to bind to Bet v 1-binding proteins in sensitized nasal epithelium rapidly and is transported through a lipid raft and caveolar-dependent process before binding to mast cells in the lamina propria [30–32]. This process is not observed with many other allergens. This indicates that the inflammatory processes of different allergens to which the epithelium is sensitized are somewhat different. However, further research is required to clarify this hypothesis.

To the best of our knowledge, no previous study has investigated such a large sample size in an investigation of the relationship between allergy and sinusitis. However, there were several limitations to our study. First, because it was a cross-sectional study, we could not infer causality in our study. Second, the definitions of allergy symptoms and sinusitis in our study were obtained from questionnaire, considering that allergic rhinitis and sinusitis may be asymptomatic in poor perceivers, the conclusions obtained may be biased. Furthermore, the defined sinusitis cannot distinguish between chronic sinusitis and acute sinusitis. When compared with studies with a clear diagnosis report, our study may thus have had some biases.

## Conclusion

These findings provide support for the hypothesis of an association between allergy and sinusitis. It is likely that sensitization status could reduce the incidence of sinusitis, albeit in an antigen-specific manner.

## Data Availability

All the data of our study come from the National Health and Nutrition Examination Survey 2005–2006. Relevant data can be obtained in https://wwwn.cdc.gov/nchs/nhanes/continuousnhanes/default.aspx?BeginYear=2005.

## Abbreviations

NHANES: The National Health and Nutrition Examination Survey
NCHS: The National Center for Health Statistics
PAS group: Positive allergy symptoms group
NAS group: Negative allergy symptoms group
CI: Confidence interval
OR: Odds ratio
SAS: Sensitization group with allergy symptoms
nSAS: Non-sensitized group with allergy symptoms
SoAS: Sensitization group without allergy symptoms
NSoAS: The non-sensitized group without allergy symptoms
